# Rapid identification of COVID-19 severity in CT scans through classification of deep features

**DOI:** 10.1186/s12938-020-00807-x

**Published:** 2020-08-12

**Authors:** Zekuan Yu, Xiaohu Li, Haitao Sun, Jian Wang, Tongtong Zhao, Hongyi Chen, Yichuan Ma, Shujin Zhu, Zongyu Xie

**Affiliations:** 1 Academy for Engineering and Technology, Fudan University, Shanghai, 200433 China; 2 Department of Radiology, The First Affiliated Hospital of Anhui Medical University, No.218 Jixi Road, Hefei, Anhui 230022 China; 3 Shanghai Institute of Medical Imaging, and Department of Interventional Radiology, Zhongshan Hospital, Fudan University, No. 180 Fenglin Road, Xuhui District, Shanghai, 200032 China; 4 Department of Radiology, Tongde Hospital of Zhejiang Province, Hangzhou, 310012 China; 5 Department of Radiology, Fuyang Second People’s Hospital, Fuyang, 236000 China; 6 The First Affiliated Hospital of Bengbu Medical College, No. 287 Changhuai Road, Bengbu Anhui, 233004 China; 7 School of Geographic and Biologic Information, Nanjing University of Posts and Telecommunications, Nanjing, 210023 China

**Keywords:** COVID-19, Tomography, Pneumonia, Coronavirus, Deep learning

## Abstract

**Background:**

Chest CT is used for the assessment of the severity of patients infected with novel coronavirus 2019 (COVID-19). We collected chest CT scans of 202 patients diagnosed with the COVID-19, and try to develop a rapid, accurate and automatic tool for severity screening follow-up therapeutic treatment.

**Methods:**

A total of 729 2D axial plan slices with 246 severe cases and 483 non-severe cases were employed in this study. By taking the advantages of the pre-trained deep neural network, four pre-trained off-the-shelf deep models (Inception-V3, ResNet-50, ResNet-101, DenseNet-201) were exploited to extract the features from these CT scans. These features are then fed to multiple classifiers (linear discriminant, linear SVM, cubic SVM, KNN and Adaboost decision tree) to identify the severe and non-severe COVID-19 cases. Three validation strategies (holdout validation, tenfold cross-validation and leave-one-out) are employed to validate the feasibility of proposed pipelines.

**Results and conclusion:**

The experimental results demonstrate that classification of the features from pre-trained deep models shows the promising application in COVID-19 severity screening, whereas the DenseNet-201 with cubic SVM model achieved the best performance. Specifically, it achieved the highest severity classification accuracy of 95.20% and 95.34% for tenfold cross-validation and leave-one-out, respectively. The established pipeline was able to achieve a rapid and accurate identification of the severity of COVID-19. This may assist the physicians to make more efficient and reliable decisions.

## Background

Since December 2019, the outbreak of a new coronavirus, named novel coronavirus 2019 (COVID-19), has rapidly spread across China and other countries across the globe [1–4]. As of 19 July, 14,043,176 cases of COVID-19 with 597,583 deaths have been reported [5]. Since the World Health Organization declared the COVID-19 outbreak as a public health emergency of international concern, namely a pandemic, countries around the globe have heightened their surveillance to quickly diagnose potential new cases of COVID-19. Due to increasing outbreak of COVID-19, the early diagnosis of patients is crucial for prompt and effective in preventing and controlling of COVID-19. Presently, nucleic acid testing is generally considered as diagnostic ground truth. However, the stringent requirements of transportation and storage of COVID-19 nucleic acid kits may constitute an unsurmountable challenge for many existing transportation and hospital facilities in crisis. Moreover, the methodology, disease development stages and the method of sample collection could impact the result of nucleic acid testing [6]. The reverse transcription polymerase chain reaction (RT-PCR) could be used for identification of COVID-19, but it is difficult to identify the severity of COVID-19 patients, to predict whether the patient should be transferred to ICU or would need ventilators soon. These factors prolong the time to control the spread of COVID-19 and increase the recovery time of patients.

Chest CT, especially, high-resolution CT, is an important tool to detect the lung changes of 2019 novel coronavirus pneumonia (NCP) and to aid in evaluating the nature and extension of lesions. In a recent report, Ai et al. [7] utilized CT scans to investigate its diagnostic value and consistency in comparison with RT-PCR assay for COVID-19. It has been found that of 1014 patients, 59% had positive RT-PCR results, while 88% had positive chest CT scans which means chest CT has a high sensitivity for diagnosis of COVID-19. Hence, the Chest CT may be treated as a primary tool to detect COVID-19 in epidemic areas. Some other investigators focused on the understanding of virus infection pathogenesis by observing the imaging patterns on chest CT. Bernheim et al. [8] characterized chest CT findings in 121 COVID-19-infected patients in relationship to the time between symptom onset and the initial CT scan. Pan et al. [9] investigated the lung abnormalities by observing the changes in chest CT of patients from initial diagnosis to recovery. It was observed that the lung abnormalities on chest CT showed greatest severity approximately 10 days after initial onset of symptoms. Most of the concern in recent reports is with the diagnosis of the COVID-19 or the clinical observation during the therapeutic treatment [10–13].

Although for most COVID-19 patients, the clinical symptoms are mild and the prognosis is good, about 20% can develop into severe cases with the symptoms of pneumonia, pulmonary edema, septic shock, metabolic acidosis, acute respiratory distress syndrome or even death [14]. Therefore, the timely diagnosis, accurate assessment with the following symptomatic treatment is very important and is the key to improve the prognosis and reduce the mortality.

It is known that convolutional neural networks (CNNs) have been proved to be powerful in data mining, image classification/detection, and computer vision. Many research groups have applied deep learning methods into COVID-19 computer aided diagnosis [15–17]. But to our best knowledge, few studies were focused on the identification of severity of infected patients, although this identification is a crucial evaluation criterion to develop proper therapeutic treatment strategy.

Therefore, developing a rapid, accurate and automatic tool for COVID-19 severity screening is both an urgent and essential task, which could help physicians anticipating the need for ICU admission. Thus, to achieve an accurate and efficient COVID-19 severity diagnosis, we classified features gained from pre-trained CNNs such as Inception v3 [18], ResNet [19] and DenseNet [20] to identify the severity of COVID-19 patients in this study. Amid the crisis in hospitals and due to challenges of training a network from scratch (e.g., necessity of a large dataset), we find this approach to be more practical and reliable.

## Results

**Fig. 1:**
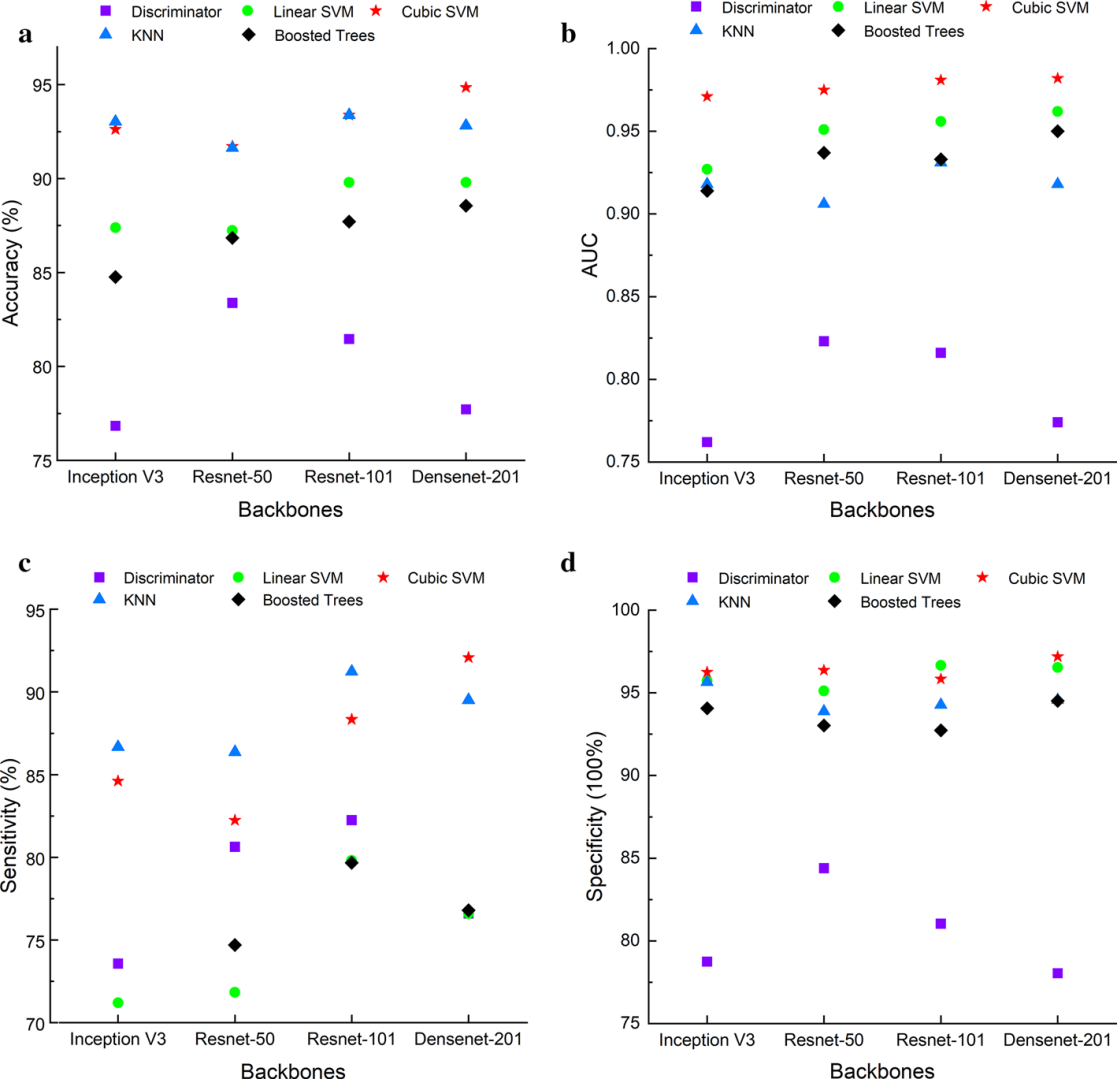
The performance of classified deep features based on holdout validation: **a** The accuracy and AUC performance; **b** The AUC performance; **c** The sensitivity performance; (d) The specificity performance

In this section, three experiments are performed to validate the feasibility of the proposed method. These include holdout validation, *k*-fold cross-validation, and leave-one-out validation schemes. All the experiments were implemented in Matlab 2019b with Intel Xeon Gold 6252 @2.1 GHz CPU and 16 GB RAM environment, and five classification methods were trained. These classifiers included linear discriminant, linear SVM (support vector machine), cubic SVM, K-nearest neighbor (KNN), and AdaBoost decision trees. We used the default parameters for these classification methods and no extra optimization was performed. For holdout validation experiment, 80% of deep features were randomly selected as training dataset and the remaining 20% were used for testing. Figures 1 reports the results of holdout validation in terms of accuracy, AUC, sensitivity and specificity. Obviously, the linear discriminator (purple square) cannot achieve a good accuracy and AUC performance, while the AdaBoost decision trees (black diamond) and linear SVM (green circle) perform worse than other three classifiers with respect to sensitivity and specificity values. Among five classification methods, the cubic SVM (red star) performs the best for all cases with respect to accuracy, AUC, sensitivity and specificity values. We may also observe that all deep models with cubic SVM classifier are able to achieve favorable results while the DenseNet-201 model does contribute to the best results for most cases.

In another series of experiments, tenfold cross-validation was performed to validate the performance of severity classification for four deep learning models. The deep features were split into tenfolds. For each fold, nine out-of-fold observations were used to train the classifier and the remaining fold was used to assess the trained classifier. The average test error over all folds was considered as the final result. The performances of cubic SVM with tenfold cross-validation are reported in Table 1. We may observe that four deep learning models are able to achieve high accuracy values in identifying the severe and non-severe COVID-19 cases with accuracy over 91.9%. Among the four topologies, the DenseNet-201 which is believed to be more representative and semantically correct in extracting features, contributes to the best result with an accuracy of 95.2% and the AUC performance of 0.99. While the ResNet-101 which contains more layers outperforms ResnNet-50 in both accuracy and AUC performance. This may because that the deep layers have better capacity in representing subtle changes like ground-glass opacities in chest CT. In addition, the DenseNet-201 also achieves the best performance for sensitivity and specificity, which increases about 7% for sensitivity and improves from 95.84% to 96.87% for specificity, respectively, when compared with Inception-V3. Generally, the high sensitivity means a high positive result (also known as the “true positive” rate) which may be more important than specificity in disease diagnosis under epidemic conditions. Thus, the DenseNet-201 is more preferable than other three architectures for severity identification of COVID-19 in CT scans.

**Table 1:** Performance of different deep learning models with cubic SVM based on tenfold cross-validation

Backbone	Accuracy (%)	AUC	Sensitivity (%)	Specificity (%)
Inception-V3 [18]	91.91	0.97	84.96	95.84
ResNet-50 [19]	92.45	0.98	85.85	96.07
ResNet-101 [19]	94.24	0.98	89.02	96.06
DenseNet-201 [20]	*95.20*	*0.99*	*91.87*	*96.87*

The highest performance value is in italics

To further investigate the performance, the leave-one-slide-out validation strategy was performed. In this experiment, all 728 deep feature vectors (out of 729) were fed to train different classifiers and the remaining one sample was used to test. This strategy is the logical extreme of *k*-fold cross-validation method. This leads to the reduced overall variability and bias than the validation-set method. The accuracy results of leave-one-out validation strategy for different deep learning methods and classifiers are shown in Tables 2, 3. We may observe that the DenseNet-201 features with cubic SVM still perform the best with a classification accuracy of 95.34%.

**Table 2:** Classification accuracy performance of deep features based on leave-one-out strategy (%)

Backbone	Discriminant	Linear SVM	Cubic SVM	KNN	Boosted trees
Inception-V3	78.88	86.15	92.47	93.69	85.32
ResNet-50	*80.52*	89.03	93.02	92.73	87.11
ResNet-101	78.74	90.53	93.69	93.96	89.44
DenseNet-201	65.95	90.53	*95.34*	*94.24*	*89.57*

The highest performance value is in italics

**Table 3:** Feature extraction time for feature extraction

Feature extraction time	Inception-V3	ResNet-50	ResNet-101	Densenet-201
Per image (s)	0.398	0.265	0.563	0.786

**Fig. 2:**
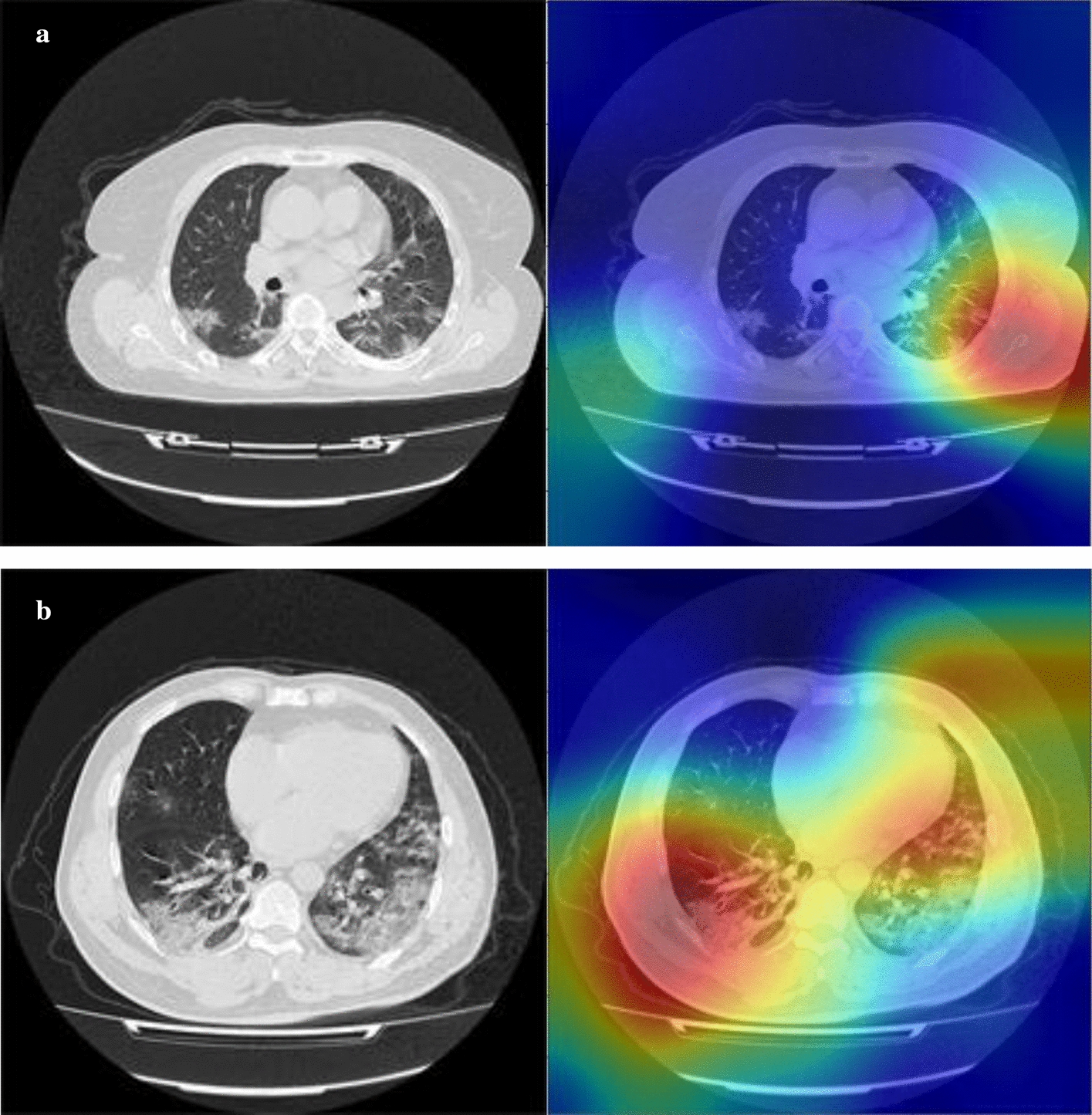
Two sample attention maps from the last ‘pooling’ layer in DenseNet-201. Whereas the attention seems to be generally rather non-exclusive, it may sometimes not contribute to human interpretation. Restricting deep feature learning or extraction to the lung regions is expected to improve the interpretability of the attention maps

To make the deep features of our pipeline more explainable, the attention maps from the last ‘pooling’ layer in DenseNet-201 are depicted in Fig. 2. These attention maps may show the discriminant 2D locations for the identification of COVID-19 severity based on consecutive convolutional filtering and undersampling. These attention spots may or may not correspond to expert understanding. One factor that may improve the attention is to restrict the filtering to lungs via masking CT scans through lung segmentation. In addition, we also give the implement time for deep feature extraction and prediction in Tables 4, 5, respectively.

**Table 4:** Feature extraction time for testing

Test time	Discriminant	Linear SVM	Cubic SVM	KNN	Boosted trees
Per image (s)	0.0421	0.0402	0.0403	0.0453	0.0564

**Table 5:** The clinical data analysis of COVID-19 confirmed patients

Characteristics	Total cases (*N* = 202)	Non-severely ill (*N* = 161)	Severely ill (*N* = 41)	*P*
Clinical data				
Gender (male)	110 (54.5%)	82 (50.9%)	28 (68.3%)	0.01
Age (mean ± S.D., year)	46.4 ± 15.554	43.7 ± 14.600	57.0 ± 14.782	< 0.001
Coexisting Illness	53 (26.2%)	30 (18.6%)	23 (56.1%)	< 0.001
Symptoms				
Fever	157 (77.7%)	118 (73.3%)	39 (95.1%)	0.523
Cough	109 (54.0%)	82 (50.9%)	27 (65.9%)	0.087
Sputum production	48 (23.8%)	38 (23.6%)	10 (24.4%)	0.916
Sore throat	17 (8.4%)	15 (9.3%)	2 (4.9%)	0.361
Nausea/headache	11 (5.4%)	9 (5.6%)	2 (4.9%)	0.858
Myalgia or arthralgia	15 (7.4%)	12 (7.5%)	3 (7.3%)	0.976
Shortness of breath	16 (7.9%)	11 (6.8%)	5 (12.2%)	0.256
Others	8 (4.0%)	8 (5.0%)	0 (0%)	0.145
Laboratory findings				
Increase of CRP	129 (63.9%)	92 (57.1%)	37 (90.2%)	< 0.001
WBCs abnormality^a^	73 (36.1%)	50 (31.1%)	23 (56.1%)	0.011
Lymphocytes abnormality^a^	109 (54.0%)	76 (47.2%)	33 (80.5%)	< 0.001
Fever				
High fever	12 (5.9%)	2 (1.2%)	10 (24.4%)	< 0.001
Low fever	145 (71.8%)	116 (72.0%)	29 (70.7%)	0.867
Travel or contact history	92 (45.5)	79 (49.1%)	13 (31.7%)	0.046

Normal body temperature: 36.3 °C–37.2 °C; Normal value of CPR: 0–10 mg/l; Normal value of WBCs: 3.5–9.5 × 109/l; Normal ratio of lymphocytes: 20%–50%; High fever (≥ 39.0 °C)

^a^Increment or reduction

## Discussion

**Fig. 3:**
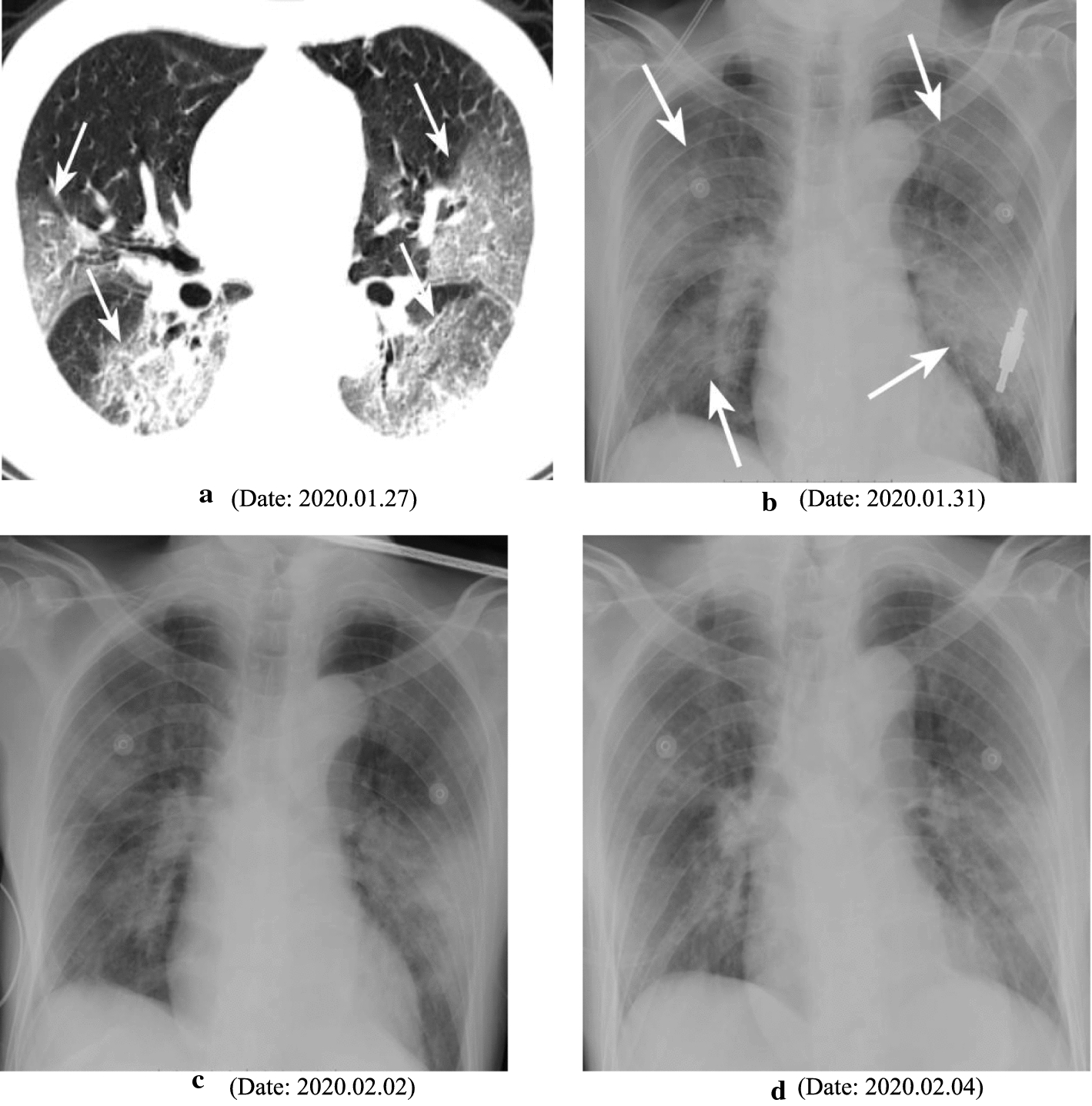
CT and DR images of a 76-year-old male with fever, cough and expectoration: **a** Chest CT scan. **b**–**d** Follow-up DR images

The COVID-19 virus, first found in Wuhan, has spread across the globe and has been formally declared as pandemic by the World Health Organization. No symptoms in the early stages of disease and the community transmission lead to the fast spread of the coronavirus (with estimated reproduction number R0 of 2.2–6.4). Since the outbreak of COVID-19, the nucleic acid testing is treated as the ground truth to identify the present of the virus. But some recent reports reveal that the accuracy of nucleic acid testing COVID-19 is about 30–50% [6]. Hence, the Chinese government has changed the diagnostic protocol to switch to CT scans for diagnosis of suspected cases [21]. Compared to the conventional X-ray, CT scans allow radiologists to inspect internal structures with much more details. Figure 3 shows the CT and DR images of a 76-year-old male COVID-19 patient with fever, cough and expectoration. It illustrates that multiple patchy regions with solid components in bilateral lung lobes can be easily detected in CT slides than in DR images. Thus, although no diagnostic test may provide complete certainty, and although this work is focused on severity identification, the CT scan seems to be an acceptable alternative diagnostic protocol to identify the COVID-19. A practical challenge that remains is the thorough disinfection of the CT machine after each scanning session is absolutely necessary.

Deep learning, which has proven to be a powerful tool in medical image processing, has been employed in COVID-19 identification or diagnosis in recent reports. Some researchers tried to use the deep model to discriminate between COVID-19 patients and bacteria pneumonia patients/healthy ones. Xu et al. [15] developed a COVID-19 screening system which can identify COVID-19, Influenza-A viral pneumonia and healthy cases. A total of 618 CT samples were collected in the study, and the proposed deep model could achieve 86.7% accuracy. Song et al. [13] proposed DRENet architecture for COVID-19 screening, which achieved 96% accuracy and 0.99 AUC among 88 COVID-19 patients with 777 images and 86 healthy persons with 708 images. Li eta al. [12] exploited ResNet50 to extract deep features to identify COVID-19 and other non-pneumonia cases. The dataset was consisted of 4356 chest scans from 3322 patients, and the proposed method achieved 90% sensitivity and 96% specificity with AUC of 0.96. Some researchers used the deep model to segment or detect the interested regions. Fan et al. [15] developed a deep learning system for automatic segmentation and quantification of COVID-19-infected regions, where their proposed VB-Net achieved 91.6% ± 10% dice similarity coefficients between automatic and manual segmentations. Gozes et al. [16] proposed an AI-based automated CT image analysis tools for detection and quantification of COVID-19. Zheng et al. [22] exploited a U-net to locate the infected region whose results were fed to a 3D deep neural network (DeCoVNet) to predict the probability of COVID-19 infection.

Although a lot of efforts have been recently focused on the automatic identification of COVID-19, few studies pay attention to automatic or semi-automatic severity assessment of COVID-19 which can track and measure the disease in a quantitative way. For instance, Tang [23] employed the random forest model to assess the severity of COVID-19 CT images from 176 patients, achieving 87.5% accuracy and 0.91 AUC.

To identify the severity of COVID-19 rapidly, and to provide an efficient and accurate prognosis to guide the follow-up therapeutic treatment, the classification of deep features for severity identification was developed in this work. The experimental results demonstrate that the proposed approach has a good ability in discriminating the severe versus non-severe cases of COVID-19 with an accuracy of 95.2%, a sensitivity of 91.87% and the specificity of 96.87%, respectively. The proposed method can be applied for severity screening.

Although the proposed method shows a promising application, there are some limitations that should be mentioned: (a) The CT data were collected only from three hospitals within one province; more variable samples at different disease stages or cases from other regions should be included in our future dataset, (b) the number of training samples was rather limited, especially the severity samples (that is why we abandon the idea of training a network from scratch), and (c) only a few physicians were involved in this dataset labeling and identification; the impact of inter-observer variability should be studied when a larger dataset is curated by more radiologists to more comprehensively represent the uncertainties of COVID-19 in CT scans.

Future researches will focus on the following aspects: (a) the volume CT scans are explored to achieve a more reliable and accurate COVID-19 severity assessment by considering the overall evaluation from 3D CT data; (b) the pre-processing method will be introduced to locate or segment the interested region to avoid the confusion brought by clothes or other artifacts; (c) the deep network will be applied on the observation of CT scan of the COVID-19 patients in their remission and recovery.

## Conclusion

In summary, our study demonstrated the feasibility of classification of pre-trained deep features to assist physicians to identify the severity of COVID-19. By achieving a good performance on severe and non-severe diagnosis, the proposed pipeline may enable a rapid identification and help the physicians make more reliable decisions for treatment planning.

## Methods

### Patients

We collected the CT volume data of 202 COVID-19 patients from three hospitals in Anhui Province, China, captured from January 24 to February 12, 2020. These cases were provided by the First Affiliated Hospital of Bengbu Medical college, the First Affiliated Hospital of Anhui Medical University, and Fuyang Second People’s Hospital. All collected cases satisfied the following instructions: (a) the result of RT-PCR was positive for throat swab, and sputum or bronchoalveolar lavage (BAL) was confirmed; (b) the availability of thin slice CT images; (c) the image quality of CT image was sufficient for radiological evaluation. Then, the patients in accordance with any of the following conditions were further marked as severely ill patients:

- shortness of breath with respiratory rate no less than 30 breaths/min;
- the oxygen saturation no more than 93% in a resting state;
- partial arterial oxygen pressure (PaO2) or fractional inspired oxygen concentration (FiO_2_) no more than 300 mmHg;
- significant progression of pulmonary lesions (over 50%) within 24–48 h;
- respiratory failure with the requirement of mechanical ventilation;
- occurrence of shock;
- multiple organ failure.

The remaining patients were regarded as non-severely ill. Then, radiologists selected 729 axial slices from these 202 CT volumes to build the dataset. Finally, 41 severe cases with 246 axial slices and 161 non-severe cases with 483 axial slices were included in this dataset.

This retrospective study (enrolled medical datasets) was approved by the ethics committees of the participating hospitals.

### Clinical information

There are 110 males and 92 females (aged from 5 to 86 years) in the dataset with average age of 46.4 ± 15.5. A total of 92 cases have travel history to epidemic area or close contact history of COVID-19 patient. As many as 53 patients had underlying coexisting illness while no coexisting illness was reported for the remaining 149 cases. Most of the patients exhibited clinical symptoms or physical findings such as fever, cough, sputum production, sore throat, nausea or headache, myalgia or arthralgia and shortness of breath. Specifically, 14 patients had high fever and 169 patients had low fever. Among most cases, the abnormal biomedical indicators or laboratory findings were generally reported, such as the abnormality of white blood cells (WCBs), neutrophils, lymphocytes and the increase of C-reactive protein (CRP) and nuclear cells. The details of clinical characteristics are shown in Table 5.

### Imaging protocol and analysis

**Fig. 4:**
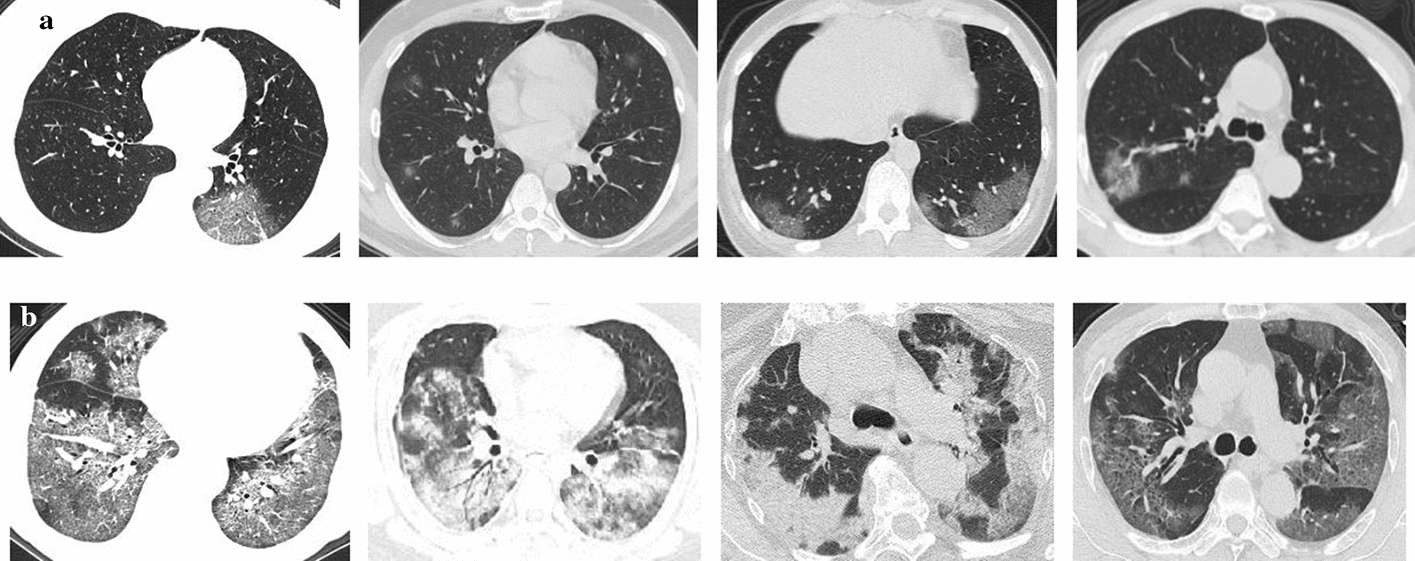
Sample CT scans of COVID-19-infected patients: **a** non-severe cases; **b** severe cases

**Fig. 5:**
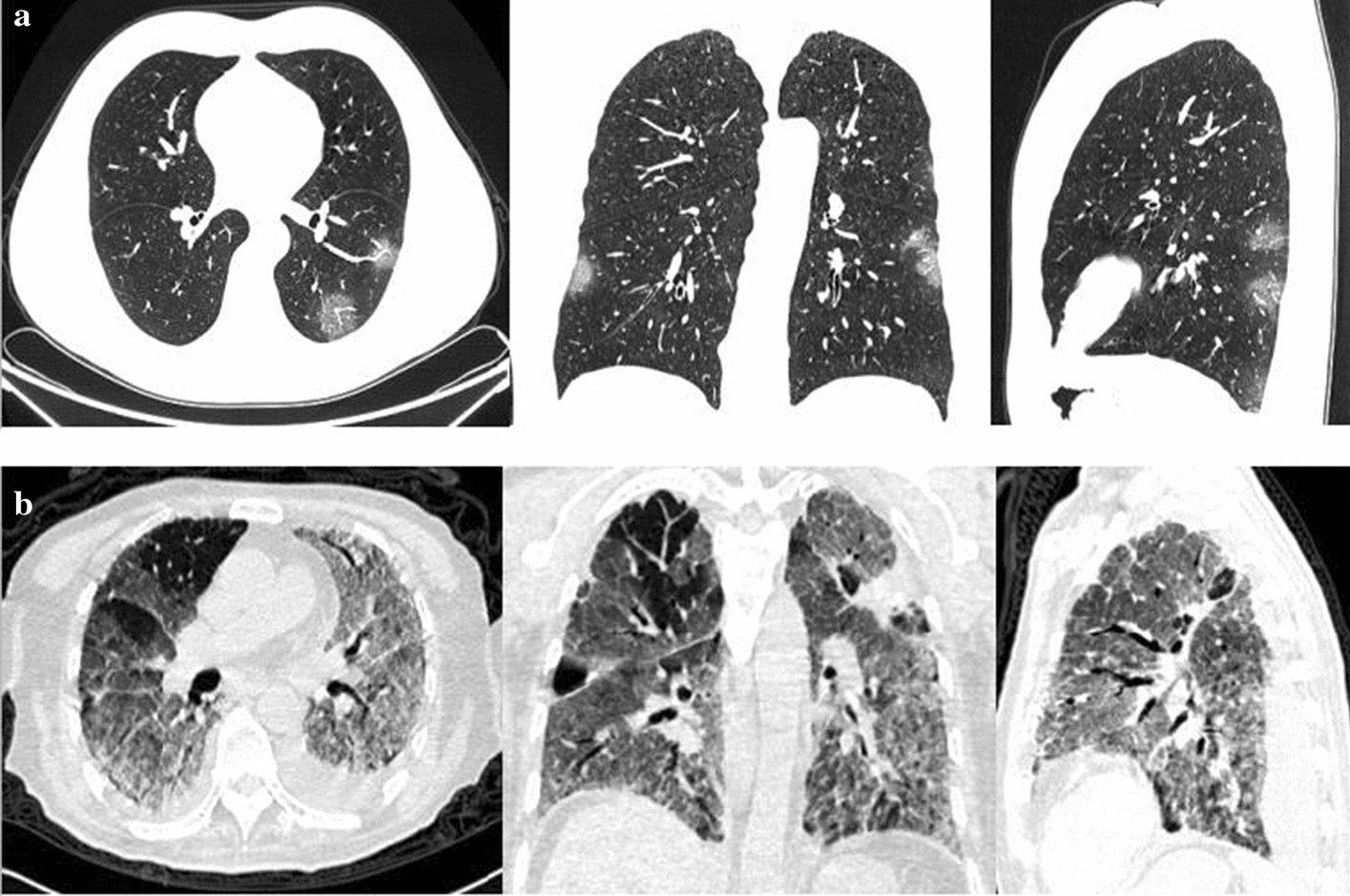
Typical examples for severe and non-severe CT chest slides in axial, sagittal and coronal views: **a** non-severe cases; **b** severe cases

In most COVID-19 cases, the bilateral incidences of consolidation, ground-glass opacities and the crazy paving pattern can be found in the lungs, where the limited or scattered nodular shadowing is observed in non-severe cases, while the flaky or widespread lesion is observed in the severe cases. Moreover, compared with non-severe cases, bronchial wall thickening, lymph node enlargement, pleural effusion, and the air bronchus-charging sign with thickened blood vessel are often observed in severe cases. Figure 4 shows some thumbnails of severe and non-severe COVID-19 chest CT scans. Figure 5 shows typical examples of severe and non-severe CT chest slides in different planes.

### Feature extraction from the pre-trained deep learning models

As training of deep networks from scratch would need a large and well-curated dataset, and the fine-tuning strategy has the advantage over the off-the-shelf when the scan labels are plentiful. Thus in this work, with limited dataset amount and labels, the off-the-shelf strategy was exploited. Specifically, the pre-trained deep models such as Inception [18], ResNet [19] and DenseNet [20] models (trained by 20.0 million images by ImageNet dataset) was exploited in this work. The image transformation in each successive layers of the DenseNet-201 network is shown in Table 6.

**Table 6:** The DenseNet-201 architectures

Layers	Output size	DenseNet-201
Convolution	112 × 112 × 64	7 × 7 conv, stride 2, padding 3
Pooling	55 × 55 × 64	3 × 3 maxpool, stride 2, padding 1
Dense block (1)	55 × 55 × 32	$$\left[ {\begin{array}{*{20}c} { 1\times 1 {\text{conv, stride 1, padding 0}}} \\ { 3\times 3 {\text{conv, stride 1, padding 1}}} \\ \end{array} } \right] \times 6$$
Transition layer (1)	55 × 55 × 128	1 × 1 conv, stride 1, padding 0
	26 × 26 × 128	2 × 2 average pool, stride 2, padding 0
Dense block (2)	26 × 26 × 32	$$\left[ {\begin{array}{*{20}c} { 1\times 1 {\text{conv, stride 1, padding 0}}} \\ { 3\times 3 {\text{conv, stride 1, padding 1}}} \\ \end{array} } \right] \times 1 2$$
Transition layer (2)	26 × 26 × 256	1 × 1 conv, stride 1, padding 0
	13 × 13 × 256	2 × 2 average pool, stride 2, padding 0
Dense block (3)	11 × 11 × 32	$$\left[ {\begin{array}{*{20}c} { 1\times 1 {\text{conv, stride 1, padding 0}}} \\ { 3\times 3 {\text{conv, stride 1, padding 1}}} \\ \end{array} } \right] \times 4 8$$
Transition layer (3)	11 × 11 × 896	1 × 1 conv, stride 1, padding 0
	5 × 5 × 896	2 × 2 average pooling stride 2, padding 0
Dense block (4)	5 × 5×32	$$\left[ {\begin{array}{*{20}c} { 1\times 1 {\text{conv, stride 1, padding 0}}} \\ { 3\times 3 {\text{conv, stride 1, padding 1}}} \\ \end{array} } \right] \times 3 2$$
Classification layer		7 × 7 global average pool
		1000D fully connected (FC-1000), softmax

The input image of dimension 224 × 224 × 3 is given to the first convolutional layer which consists of 64 kernels of size 7 × 7 with 2 stride and 3 padding. The stride is defined as the number of pixels shift by the filter in the image matrix. By convolving the image with 64 kernels, the output image obtained from the convolution layer is of size 112 × 112 × 64.

The final fully connected layer FC 1000 layer has dimension of 1000. In the proposed method, FC-1000 layer features from DenseNet-201 are extracted and fed into the various classifiers for classification task. The output of single convolutional layer is given by Eq. (1).

1 $$h_{i,j} = f\left( {\mathop \sum \limits_{k}^{P} \mathop \sum \limits_{l}^{Q} w_{k,l} x_{i + k,j + l} + b_{k,l} } \right),$$

where *h* represents the neuron output, *x* denotes the input, *w* represents the weight and *b* is the bias parameter. Here *P*, *Q* represent the size of weight parameters, *k*, *l* are parameter indices and *i*, *j* are input indices. Each convolutional layer follows rectified linear unit activation (ReLU), normalization and max pooling operations, and the ReLU is used as activation function in the DenseNet-201.

**Fig. 6:**
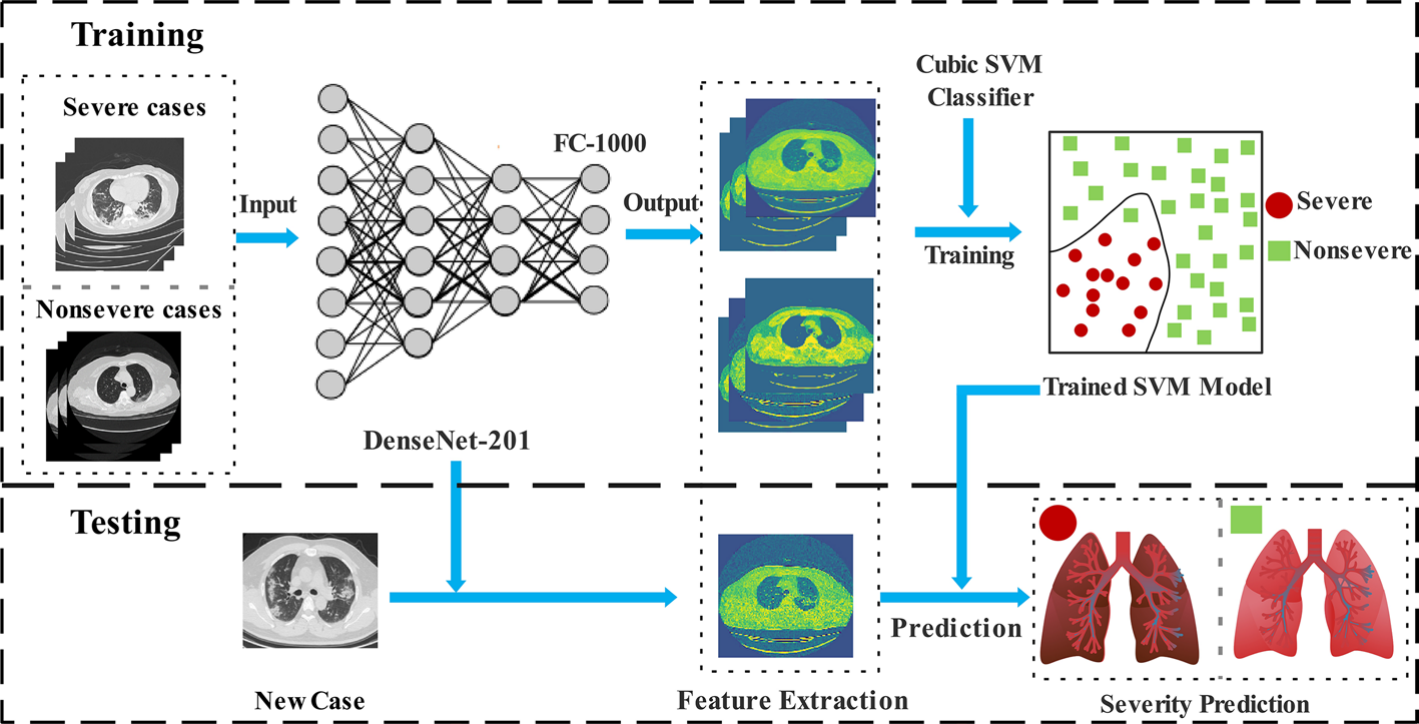
The pipeline of the proposed method

Figure 6 illustrated the details and the pipeline of the proposed method. During the training step, the pre-trained deep model (DenseNet-201) was employed to extract the deep features from COVID-19 CT scans. Subsequently, the binary SVM classifier with cubic kernel was trained to perform the classification task of severe versus not severe distinction. In the testing step, the unseen COVID-19 scan sample was input to predict the severity with the help of its deep feature and the trained classifier.

## Data Availability

Since the data used in this study include data collected in a clinical trial with patients, the data will not be shared.

## References

[1] Chan JF-W, Yuan S, Kok K-H, To KK-W, Chu H, Yang J, Xing F, Liu J, Yip CC-Y, Yip CC-Y, Poon RW-S (2020). A familial cluster of pneumonia associated with the 2019 novel coronavirus indicating person-to-person transmission: a study of a family cluster. Lancet.

[2] Li Q, Guan X, Wu P, Wang X, Zhou L, Tong Y, Ren R, Leung KS, Lau EH, Wong JY (2020). Early transmission dynamics in Wuhan, China, of novel coronavirus–infected pneumonia. N Engl J Med.

[3] Rothe C, Schunk M, Sothmann P, Bretzel G, Froeschl G, Wallrauch C, Zimmer T, Thiel V, Janke C, Guggemos W (2020). Transmission of 2019-nCoV infection from an asymptomatic contact in Germany. N Engl J Med.

[4] Giovanetti M, Benvenuto D, Angeletti S, Ciccozzi M (2020). The first two cases of 2019-nCoV in Italy: where they come from?. J Med Virol.

[5] https://www.who.int/emergencies/diseases/novel-coronavirus-2019/situation-reports.

[6] Wang S, Kang B, Ma J, Zeng X, Xiao M, Guo J, Cai M, Yang J, Li Y, Meng X. A deep learning algorithm using CT images to screen for Corona Virus Disease (COVID-19). medRxiv. 2020. 10.1007/s00330-021-07715-1 PMC7904034 33629156

[7] Ai T, Yang Z, Hou H, Zhan C, Chen C, Lv W, Tao Q, Sun Z, Xia L (2020). Correlation of Chest CT and RT-PCR Testing in Coronavirus Disease 2019 (COVID-19) in China: a Report of 1014 Cases. Radiology.

[8] Bernheim A, Mei X, Huang M, Yang Y, Fayad ZA, Zhang N, Diao K, Lin B, Zhu X, Li K (2020). Chest CT Findings in Coronavirus Disease-19 (COVID-19): relationship to duration of infection. Radiology.

[9] Pan F, Ye T, Sun P, Gui S, Liang B, Li L, Zheng D, Wang J, Hesketh RL, Yang L (2020). Time course of lung changes on chest CT during recovery from 2019 novel coronavirus (COVID-19) pneumonia. Radiology.

[10] Liu T, Huang P, Liu H, Huang L, Lei M, Xu W, Hu X, Chen J, Liu B (2020). Spectrum of chest CT findings in a familial cluster of COVID-19 infection. Radiology.

[11] Ng M-Y, Lee EY, Yang J, Yang F, Li X, Wang H, Lui MM-S, Leung B, Khong P-L (2020). Imaging profile of the COVID-19 infection: radiologic findings and literature review. Radiology.

[12] Li L, Qin L, Xu Z, Yin Y, Wang X, Kong B, Bai J, Lu Y, Fang Z, Song QJR (2020). Artificial intelligence distinguishes COVID-19 from community acquired pneumonia on chest CT. Radiology.

[13] Song Y, Zheng S, Li L, Zhang X, Zhang X, Huang Z, Chen J, Zhao H, Jie Y, Wang R. Deep learning Enables Accurate Diagnosis of Novel Coronavirus (COVID-19) with CT images. medRxiv. 2020. 10.1109/TCBB.2021.3065361 PMC8851430 33705321

[14] Li K, Wu J, Wu F, Guo D, Chen L, Fang Z, Li C (2020). The clinical and chest CT features associated with severe and critical COVID-19 pneumonia. Invest Radiol.

[15] Xu X, Jiang X, Ma C, Du P, Li X, Lv S, Yu L, Chen Y, Su J, Lang G. Deep learning system to screen coronavirus disease 2019 pneumonia. arXiv preprint arXiv:200209334. 2020. 10.1016/j.eng.2020.04.010 PMC7320702 32837749

[16] Gozes O, Frid-Adar M, Greenspan H, Browning PD, Zhang H, Ji W, Bernheim A, Siegel E. Rapid AI Development Cycle for the Coronavirus (COVID-19) Pandemic: Initial Results for Automated Detection & Patient Monitoring using Deep Learning CT Image Analysis. arXiv preprint arXiv:200305037. 2020.

[17] Shan F, Gao Y, Wang J, Shi W, Shi N, Han M, Xue Z, Shen D, Shi Y. Lung Infection Quantification of COVID-19 in CT Images with Deep Learning. arXiv preprint arXiv:200304655. 2020.

[18] Szegedy C, Vanhoucke V, Ioffe S, Shlens J, Wojna Z. Rethinking the inception architecture for computer vision. In: Proceedings of the IEEE conference on computer vision and pattern recognition. 2016. p. 2818–26.

[19] He K, Zhang X, Ren S, Sun J. Deep residual learning for image recognition. In: Proceedings of the IEEE conference on computer vision and pattern recognition. 2016. p. 770–8.

[20] Huang G, Liu Z, Van Der Maaten L, Weinberger KQ. Densely connected convolutional networks. In: Proceedings of the IEEE conference on computer vision and pattern recognition. 2017. p. 4700–8.

[21] https://www.nytimes.com/2020/02/09/world/asia/china-coronavirus-tests.html.

[22] Zheng C, Deng X, Fu Q, Zhou Q, Feng J, Ma H, Liu W, Wang XJM (2020). Deep learning-based detection for COVID-19 from chest CT using weak label. medRxiv.

[23] Tang Z, Zhao W, Xie X, Zhong Z, Shi F, Liu J, Shen D. Severity Assessment of Coronavirus Disease 2019 (COVID-19) Using Quantitative Features from Chest CT Images. arXiv preprint arXiv:200311988. 2020.

